# Nephrocystin-1 Forms a Complex with Polycystin-1 via a Polyproline Motif/SH3 Domain Interaction and Regulates the Apoptotic Response in Mammals

**DOI:** 10.1371/journal.pone.0012719

**Published:** 2010-09-14

**Authors:** Claas Wodarczyk, Gianfranco Distefano, Isaline Rowe, Massimiliano Gaetani, Barbara Bricoli, Mordi Muorah, Andrea Spitaleri, Valeria Mannella, Piero Ricchiuto, Monika Pema, Maddalena Castelli, Ariel E. Casanova, Luca Mollica, Manuela Banzi, Manila Boca, Corinne Antignac, Sophie Saunier, Giovanna Musco, Alessandra Boletta

**Affiliations:** 1 Division of Genetics and Cell Biology, Dulbecco Telethon Institute at Dibit, San Raffaele Scientific Institute, Milan, Italy; 2 Biomolecular NMR Laboratory, Dulbecco Telethon Institute at Dibit, San Raffaele Scientific Institute, Milan, Italy; 3 INSERM, U-574, Hôpital Necker-Enfants Malades, Paris, France; 4 Université Paris Descartes, Paris, France; 5 AP-HP, Department of Genetics, Hôpital Necker-Enfants Malades, Assistance Publique–Hôpitaux de Paris, Paris, France; Johns Hopkins School of Medicine, United States of America

## Abstract

Mutations in *PKD1*, the gene encoding for the receptor Polycystin-1 (PC-1), cause autosomal dominant polycystic kidney disease (ADPKD). The cytoplasmic C-terminus of PC-1 contains a coiled-coil domain that mediates an interaction with the *PKD2* gene product, Polycystin-2 (PC-2). Here we identify a novel domain in the PC-1 C-terminal tail, a polyproline motif mediating an interaction with Src homology domain 3 (SH3). A screen for interactions using the PC-1 C-terminal tail identified the SH3 domain of nephrocystin-1 (NPHP1) as a potential binding partner of PC-1. NPHP1 is the product of a gene that is mutated in a different form of renal cystic disease, nephronophthisis (NPHP). We show that *in vitro* pull-down assays and NMR structural studies confirmed the interaction between the PC-1 polyproline motif and the NPHP1 SH3 domain. Furthermore, the two full-length proteins interact through these domains; using a recently generated model system allowing us to track endogenous PC-1, we confirm the interaction between the endogenous proteins. Finally, we show that NPHP1 trafficking to cilia does not require PC-1 and that PC-1 may require NPHP1 to regulate resistance to apoptosis, but not to regulate cell cycle progression. In line with this, we find high levels of apoptosis in renal specimens of NPHP patients. Our data uncover a link between two different ciliopathies, ADPKD and NPHP, supporting the notion that common pathogenetic defects, possibly involving de-regulated apoptosis, underlie renal cyst formation.

## Introduction

Autosomal dominant polycystic kidney disease (ADPKD) is a frequent genetic disease affecting 1/1000 people characterized by renal cyst formation. Mutations in two genes can cause ADPKD: *PKD1* and *PKD2* [1]. The first is mutated in the majority of the cases (85%), and it encodes a large (∼520 kDa) plasma membrane receptor, Polycystin-1 (PC-1).

PC-1 has a very large extracellular domain comprised of a novel combination of protein-protein interaction domains, 11 transmembrane domains and a short intracellular C-terminus containing a coiled-coil motif that mediates an interaction with the *PKD2* gene product, Polycystin-2 (PC-2) [2] [3]. The precise function of the PC-1/2 complex is largely unclear [4]. The complex localizes to cell-cell junctions [5], focal adhesions [6] and the primary cilia in renal epithelial cells [7].

Here, we report that the C-terminus of PC-1 contains at least one polyproline domain that is able to mediate an interaction with Src-homology 3 domains (SH3). We show that PC-1 interacts with the SH3 domain of Nephrocystin-1 (NPHP1), the product of the *NPHP1* gene mutated in nephronophthisis, an autosomal recessive disease characterized by a small cyst formation at the corticomedullary junction of the kidney [8], [9] [10].

NPHP1 is a cytoplasmic adaptor molecule containing a putative coiled-coil domain and an SH3 domain whose function is still unknown. Similar to PC-1, NPHP1 localizes to cell-cell junctions, cilia and cell-matrix adhesion sites [11] [12] [13]. We provide evidence of a physical and functional interaction between the products of these two genes, which are mutated in two distinct renal ciliopathies, polycystic kidney disease and nephronophthisis, supporting the notion that the molecular mechanism underlying cyst formation shares common pathogenetic dysfunctions in different diseases.

## Results

### A polyproline motif in the PC-1 C-tail interacts with the SH3 domain of NPHP1

**Figure 1:**
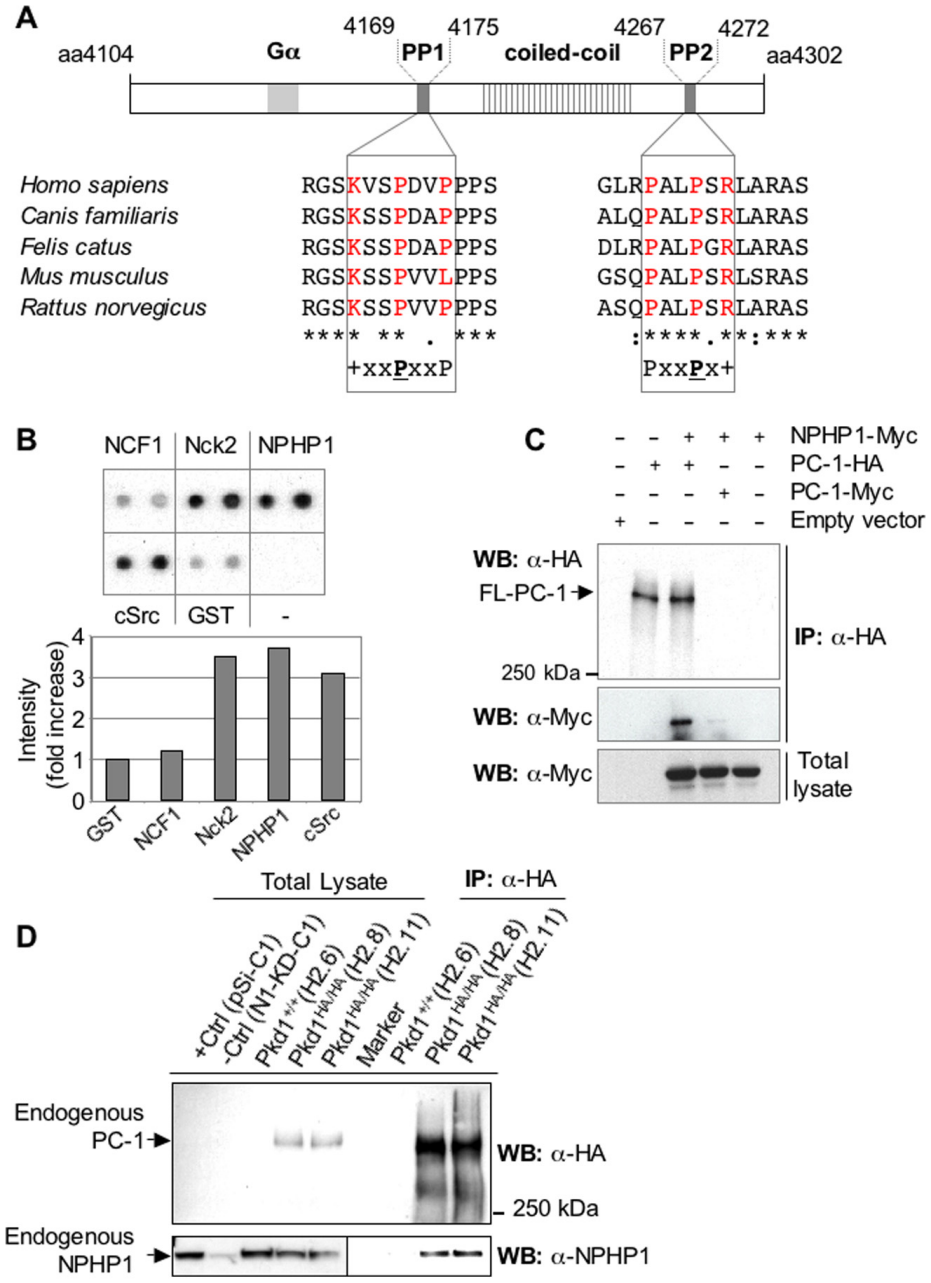
The PC-1 C-terminus contains polyproline motifs that interact with the SH3 domain of NPHP1. **A.** Sequence alignment of the two putative polyproline domains (PP1 and PP2) in the C-terminus of PC1 from different species. Asterisks indicate the conserved residues. The residues of the polyproline consensus motif are in red. **B.** The C-terminal tail of PC-1 fused to poly-HIS tag (HIS-PC-1-CT) and expressed in bacteria was incubated in the presence of membranes containing several SH3-domains fused to GST and spotted in duplicate (TranSignal™ SH3 Domains, Panomics). α-HIS antibodies were used to detect HIS-PC-1-CT that interacted with GST-SH3 domains. Quantification of the intensities (bottom histograms) showed an association with NPHP1, Nck2 and cSrc, but not with NCF1 nor GST. **C.** Full-length Myc-NPHP1 was co-expressed in HEK293 cells with full-length PC-1-HA or full-length PC-1-Myc. Immunoprecipitation using α-HA antibodies resulted in the co-immunoprecipitation of NPHP1-Myc specifically in the presence of PC-1 immunoprecipitation. **D**. MEFs derived from a recently described mouse model expressing tagged-endogenous PC-1 [16] were used to immunoprecipitate endogenous HA-tagged PC-1. Cells expressing the wild-type untagged PC-1 (*Pkd1* ^+/+^) served as a negative control for the immunoprecipitation. Cell lysates from parental MDCK cells (+Ctrl) or NPHP1–silenced cells (-Ctrl) [32] served as a control for NPHP1 western blot. Endogenous NPHP1 and PC-1 co-immunoprecipitated.

Bioinformatic analysis of the PC-1 sequence, using two public web sites (2009) (http://scansite.mit.edu and http://cbm.bio.uniroma2.it/SH3-Hunter), identified two putative SH3-interacting polyproline domains in its C-terminus, PP1 (K_4169_VSPDVP_4175_) and PP2 (P_4267_ALPSR_4272_). The last had been previously noted in the murine sequence, but its role was never investigated [14]. PP1, corresponding to the classical type I consensus sequence (R/KxxPxxP), had a very low prediction score (∼0.3), whereas PP2, corresponding to the type II (PxxPxR/K) consensus, (Figure 1A), had a high prediction score (0.97) [15].

To test if the C-terminus of PC-1 (aa 4132-4302) is able to interact with SH3 domains, we performed a screen using an overlay assay system (TranSignal™ SH3 Domains, Panomics) that allows for the simultaneous screening of 152 SH3 domains for possible interactions. We identified several potential binding partners (Figure 1B) suggesting that the C-terminal tail of PC-1 is able to interact with SH3 domains. Notably, the SH3 domain of NPHP1 (NPHP1-SH3) was identified.

PC-1 and NPHP1 localize to identical subcellular compartments and cause diseases that share common features. However, these two proteins were never reported to associate in a complex. We used a series of novel tools generated in our lab to investigate their possible interaction.

First, vectors expressing Myc-tagged NPHP1 and HA-tagged full-length PC-1 were transiently co-transfected into HEK293 cells. Co-immunoprecipitation with α-HA antibodies revealed a specific association of the two proteins when overexpressed in cells (Figure 1C). Furthermore, immunofluorescence analysis revealed a partial co-localization of the two molecules in intracellular patches (Figure S1A).

Because overexpression of molecules might force interactions that do not typically exist, we next tested if the endogenous PC-1 and NPHP1 are indeed able to form a complex in physiological conditions. To this end, we used mouse embryonic fibroblasts (MEFs) derived from a recently described mouse model in which a tag was inserted into the *Pkd1* gene resulting in the expression of a tagged endogenous PC-1 (*Pkd1* ^HA/HA^) [16]. These cells express normal levels of fully-functional PC-1, of which detection is greatly enhanced by the addition of a tag. This feature allowed us to use wild-type (*Pkd1* ^+/+^) cells as an optimal control. Immunoprecipitation of the endogenous tagged PC-1 from two independent cell lines resulted in the co-immunoprecipitation of the endogenous NPHP1 (Figure 1D), while no NPHP1 pull-down could be detected in wild-type (*Pkd1* ^+/+^) MEFs, demonstrating its specificity. Similar results were generated by using a cell line derived from mice carrying a different tag (*Pkd1* ^Myc/Myc^, Figure S1B). Collectively, these data demonstrate the existence of a complex containing both PC-1 and NPHP1.

### The second polyproline motif (PP2) interacts with NPHP1-SH3 domain

**Figure 2:**
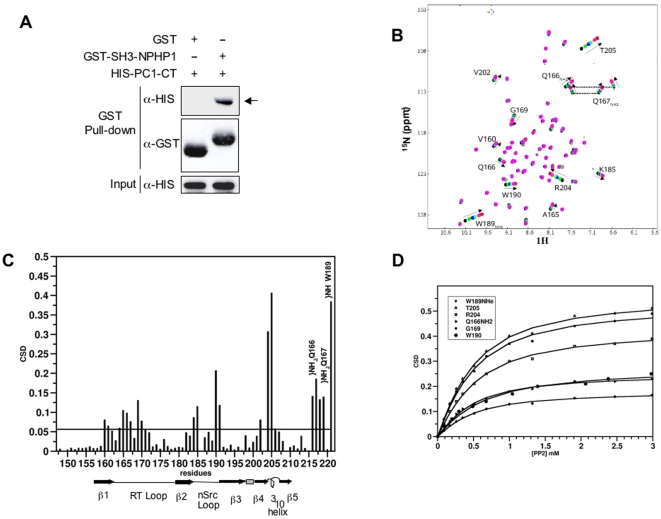
PC-1/NPHP1 interaction is mediated by a polyproline motif/SH3 domain. **A.** GST pull-down assays were performed between the GST-NPHP1-SH3 and the HIS-PC-1-CT. NPHP1-SH3, but not GST alone, precipitated HIS-PC-1-CT. **B** ^1^H-^15^N HSQC spectra of NPHP1-SH3 during the titration with peptPP2 (0, 0.5, 1, 1.5, 2, 3, 4 equivalents). The starting and ending points of the titration are represented in black and red, respectively. The observed changes are a continuous function of the amount of added peptide, indicating that the binding is in the fast exchange limit on the NMR time scale. Residues with chemical shift changes >0.05 ppm are explicitly labeled. **C** Distribution of the average backbone amide chemical shift differences (CSD) within NPHP1-SH3 (0.3 mM) upon addition of a four-fold excess of peptPP2. Secondary structure elements are shown according to NPHP1-SH3 structure (1S1N). **D**. Weighted average of ^1^H and ^15^N chemical-shift changes of selected NPHP1-SH3 residues as a function of added peptPP2.

To test if complex formation is directly mediated by the putative PC-1 polyproline motifs and NPHP1-SH3 domain, we performed glutathione s-transferase (GST) pull-down assays. Using the GST-fused SH3 domain of NPHP1 (GST-NPHP1-SH3) and the histidine tagged C-terminus of PC-1, we found that these two domains are indeed able to interact *in vitro* (Figure 2A and Figure S1C), suggesting that the C-tail of PC-1 contains motifs able to mediate an interaction with the SH3 domain of NPHP1.

**Figure 3:**
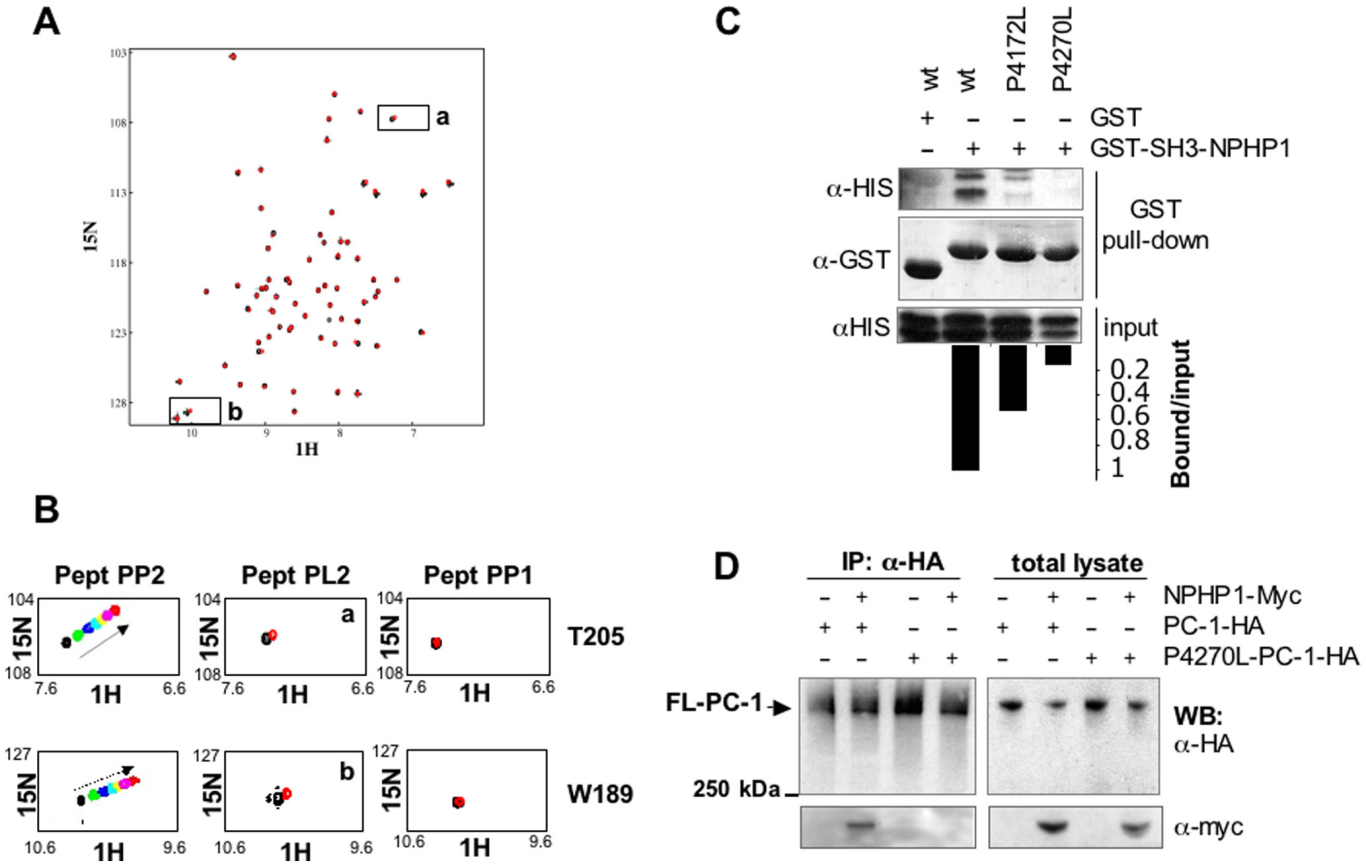
The polyproline domain 2 (PP2) of PC-1, but not the polyproline domain 1 (PP1), interacts with the SH3 domain of NPHP1 in a canonical manner. **A.** ^1^H-^15^N HSQC spectra of NPHP1-SH3 with (red) and without (black) peptPL2. The addition of the peptide does not cause any peak displacement indicating that mutation of the proline in the polyproline motif destroys the binding. **B.** Insets derived from the spectra in 2B, 3A or S1A relative to aminoacids T205 or W189 observed in the presence of peptPP2 (left), peptPL2 (middle) or peptPP1 (right) show that while these amino acids are displaced in the presence of peptPP2, only minimal movement can be observed upon addition of peptPL2 and no movement is detected with peptPP1. **C.** The experiment was carried out as described in **2A** but using HIS-PC-1-CT or mutant forms with Proline 4172 (PP1) or Proline 4270 (PP2) replaced with Leucine. **D**. Full-length Myc-tagged NPHP1 (NPHP1-Myc) was co-expressed in Hek293 cells with either the wild-type form of PC-1-HA or a mutant form carrying a leucine instead of a proline at residue 4270 (P4270L-PC-1-HA). Immunoprecipitation using α-HA antibodies revealed that this single amino acid change completely abolishes interaction with NPHP1-Myc.

Next, we performed binding assays of synthetic peptides corresponding to stretches of aminoacids derived from PC-1 C-tail, containing either PP1 (peptPP1) or PP2 (peptPP2) (Table S1), to NPHP1-SH3 using two-dimensional ^1^H-^15^N NMR. A discrete set of chemical shift changes was observed only in titrations of peptPP2 into ^15^N NPHP1-SH3 (Figure 2B and C). We estimated a dissociation constant of 0.3 +/- 0.02 mM (Figure 2D). Similar values were obtained by isothermal titration calorimetry (ITC) (Figure S3A). Importantly, a peptide corresponding to aminoacids 4264-4277 in which P4270 was substituted with a leucine (peptPL2, Table S1) completely abolished complex formation as assessed by the lack of peak displacements in ^15^N HSQC titrations (Figure 3A and B), demonstrating the specificity of the interaction. No shifts were observed upon addition of peptPP1 or peptPL1 (in which the central proline of PP1 was mutated into leucine), implying that PP1 is not able to interact with NPHP1-SH3 domain (Figure 3B and S2A). In line with the NMR data, GST pull-down experiments between the NPHP1-SH3 domain and the PC-1 C-tail revealed that the *in vitro* binding between the two molecules was completely abrogated when a proline to leucine substitution was inserted into P4270 of PC-1 (Figure 3C). Most importantly, co-immunoprecipitation assays in HEK293 cells of wt-NPHP1 with the P4270L mutant full-length PC-1 showed that this single amino acid change completely abrogates the interaction between the two full-length molecules (Figure 3D). Surprisingly, the PC-1-HIS C-tail carrying a proline to leucine substitution in P4172 showed a reduction in the binding capability to NPHP1-SH3 domain (Figure 3C), despite the fact that peptPP1 is completely unable to cause any peak displacement in two-dimensional ^1^H-^15^N NMR assays titrations(Figure 3B, right). These data might suggest that, although PP1 is unlikely to be directly involved in the binding to NPHP1-SH3, its mutation might cause changes in the tertiary structure of PC-1 C-tail able to influence the strength of binding of PP2.

Collectively, these data demonstrate that PP2 is responsible for the interaction with the SH3 domain of NPHP1, and that this interaction, albeit weak when tested on isolated peptides, is specific and essential for complex formation.

### Mapping of the interaction and binding specificity

**Figure 4:**
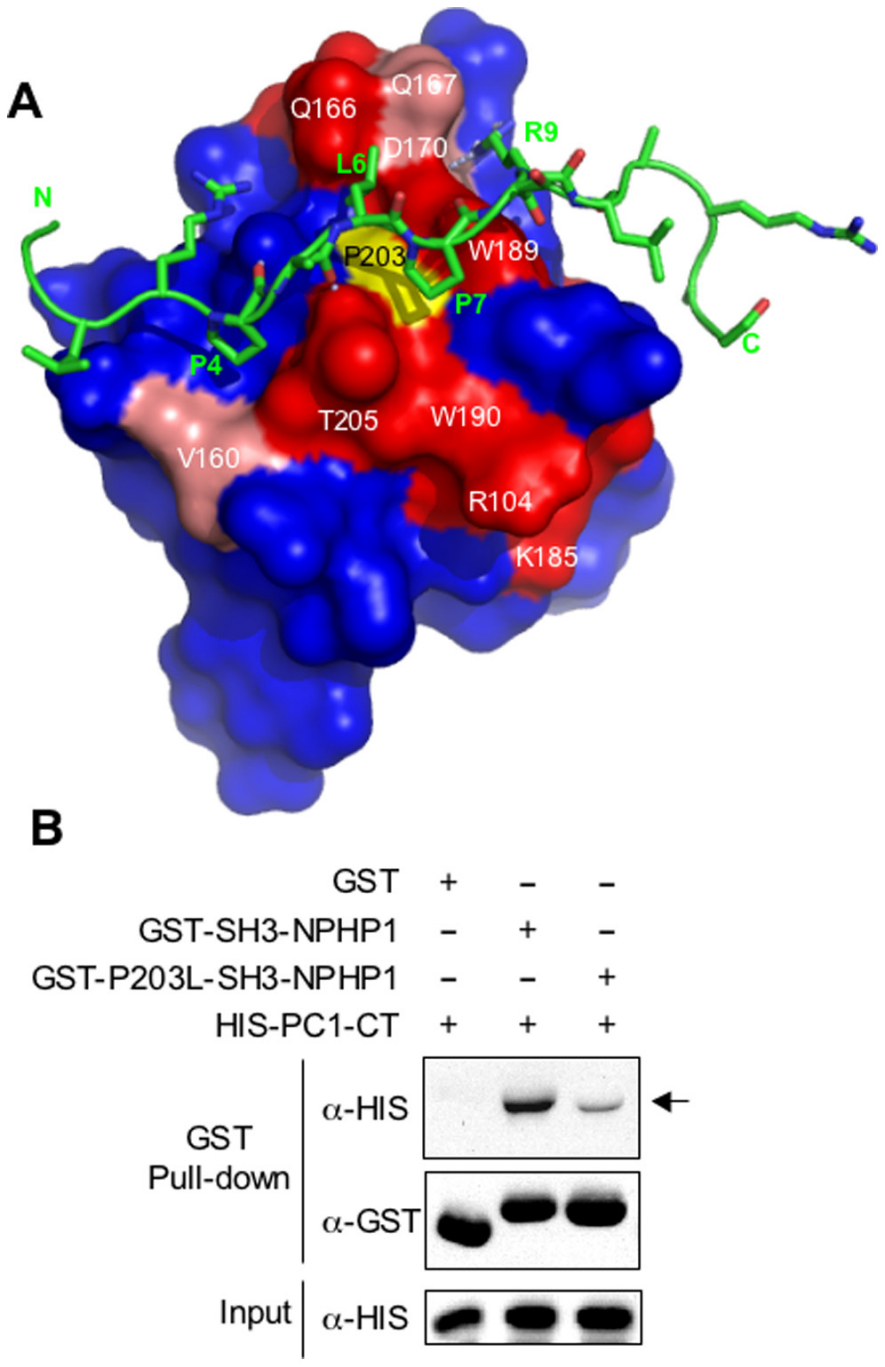
A P203L mutation in the SH3 domain of NPHP1 prevents the interaction with PC-1. **A** Model of NPHP1-SH3 (PDB code: 1S1N, surface representation) in complex with peptPP2 (represented with sticks). NPHP1-SH3 residues with the highest NH chemical shifts are highlighted in red (Δ∂>0.1ppm) and pink (0.05<Δ∂<0.1). The surface of P203 is highlighted in yellow. **B.** GST pull-down assays using either the wild-type or the mutant SH3 domain of NPHP1 (P203L) fused to GST were performed as in Figure 1C and D. The interaction is impaired in the presence of the mutant NPHP1.

To identify the interaction surface, residues with significant chemical shift differences (CSD >0.05 ppm) (Figure 2B) were mapped onto the three-dimensional structure of NPHP1-SH3 (1S1N). These residues constitute the classical polyproline binding groove, including amino acids located on the RT loop, the n-Src loop and the 3^10^ helix (V160, A165, Q166, Q167, G169, D170, K185, W189, W190, I191, V202, R204, and T205) (Figure 4A and Figure S3B).

To further characterize the specificity of the interaction, we analyzed the effect of a previously described NPHP1-SH3 mutation (P203L) on the binding to PC-1 [17]. Importantly, this mutation did not affect fold stability, as assessed both by circular dichroism thermal denaturation melting curves (T_m_ **∼**80°C) and by NMR spectroscopy (Figure S2B). However, the mutation strongly affected its function. In fact, GST pull-down assays with the C-terminus of PC-1 showed that P203L mutation impaired binding (Figure 4B). Accordingly, NMR titrations with peptPP2 showed that P203L abolished the interaction as no peak displacement was observed upon addition of peptPP2 (Figure S2C).

### Model of the interaction

We used the experimental data (chemical shift mapping and mutagenesis) to generate a model of the interaction docking peptPP2 on the known structure of NPHP1-SH3 using the HADDOCK strategy [18]. The peptide interacted with the classical SH3 binding groove adopting a type-II orientation (Figure 4A) whereby peptPP2-P4 packed against Y206, while peptPP2-L6 and peptPP2-P7 fit inside the hydrophobic pocket formed by Y206, F163, W189 and P203 (Figure 4A and Figure S3B). Importantly, the carbonyl backbone of peptPP2-P7 made a hydrogen bond with the indole NH of W189. Binding specificity was further conferred by a bifurcated electrostatic interaction between the guanidinium group of peptPP2-R9 and the backbone carbonyl of G167 and the carboxylate of D170. Finally, peptPP2-R3 created a salt-bridge with the carboxyl group of D162 (Figure S3B). Collectively, the model of the complex recapitulates the classical interaction pattern observed between SH3 domains and type II polyproline motifs, providing further evidence that PP2 is a *bona fide* polyproline domain.

### PC-1 and NPHP1 cooperate to regulate apoptosis, but not proliferation

Finally, we investigated the biological significance of the PC-1/NPHP1 interaction. Immunofluorescent staining using an α-NPHP1 antibody revealed that NPHP1 localized to the base of the cilia in both *Pkd1* ^+/+^ and *Pkd1* ^−/−^ MEFs (Figure S4A). Next, we used a mouse model carrying a *Pkd1*-floxed allele [16] crossed with a mouse harboring a kidney-specific Cre recombinase (Ksp-Cre) [19]. This resulted in massive renal cystogenesis, as previously reported [20], and the cystic epithelial cells showed intact cilia formation. We subsequently analyzed the distribution of NPHP1 and found that NPHP1 still localized to cilia in the cystic kidneys, similar to the distribution observed in normal kidneys (Figure S4B). We conclude that trafficking of NPHP1 to cilia does not depend on PC-1.

**Figure 5:**
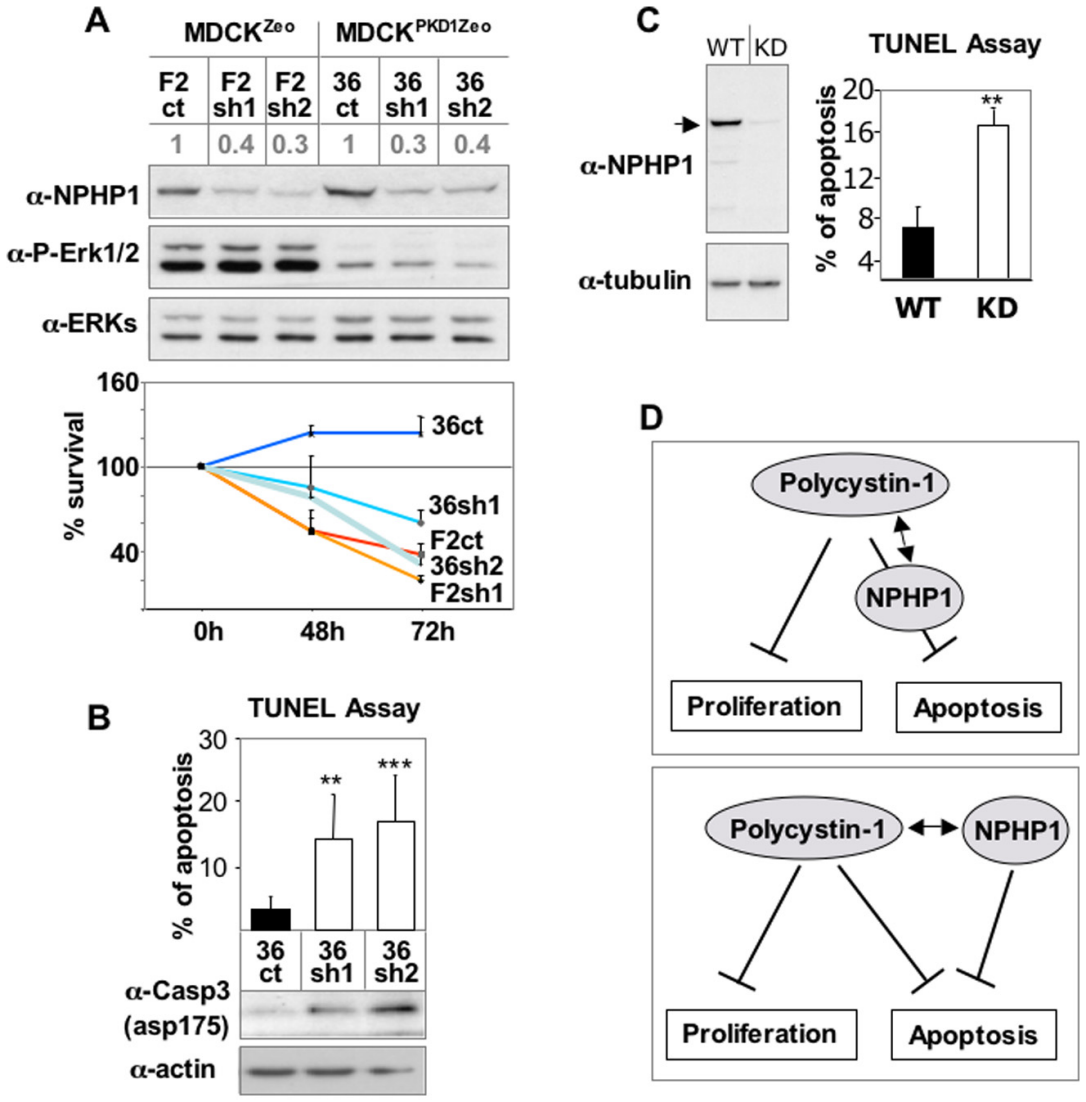
PC-1 and NPHP1 functionally interact. **A**. Top. MDCKII control cells (F2) and or over-expressing PC-1 (36) were stably transfected with shRNA against NPHP1 (sh1 and 2) or control shRNA (ct). Several clones were isolated (F2ct, F2sh1, F2 sh2, 36ct, 36sh1, 36sh2) The residual level of expression of NPHP1 after silencing in the lines carrying NPHP1-specific shRNAs is indicated in grey above each lane. Cells over-expressing PC-1 showed reduced phosphoERKs that was not affected by NPHP1 silencing. Bottom. Cell viability was evaluated by cell counting at 48 and 72 hours after TNFα treatment as an apoptotic stimulus. While shRNA control cells over-expressing PC-1 (36ct) survive, cells that were silenced for NPHP1 become sensitive to apoptosis (36sh1 and 36sh2) to an extent comparable to the controls (F2ct and F2sh1). **B**. Cells were treated as in **A.** and apoptosis analyzed by TUNEL assay and cleaved caspase 3, 48 hours after treatment. Cells carrying the NPHP1-silencing showed a significant increase in the apoptotic rates. **C**. previously reported [32] MDCK cells control (WT, pSi-C1) or silenced for NPHP1 (KD, N1-KD-C1) show a considerable dicrease in NPHP1 expression (left blot). Cells were treated as in **B** and the TUNEL assay was performed to assess the apoptotic response (right graph). **D**. Two possible models derived from the results shown in this figure. PC-1 is able to inhibit both proliferation and apoptosis [21]. The regulation of proliferation by PC-1 does not require NPHP1, whereas the regulation of apoptosis is sensitive to NPHP1 silencing, suggesting that NPHP1 might act downstream (top) or in parallel (bottom) to PC-1 converging on a common functional effect on apoptosis. Statistical analysis was performed using the ANOVA test (in B) or the Student's t-Test (in C and D). **p<0.005. ***p<0.0005.

Previous studies have demonstrated that overexpression of PC-1 induces cell cycle arrest in the G0/G1 phase and resistance to apoptosis [21] [22] [4]. In order to investigate the role of NPHP1 in these functions, we used MDCK cells overexpressing PC-1, and we achieved silencing of NPHP1 by generating stable transfectants carrying a shRNA directed against canine NPHP1 (sh1 or sh2). Scrambled shRNAs served as a control (ct) (Figure 5A, top). Silencing of NPHP1 had no effect on PC-1 capability to regulate phosphorylation of ERK and cell cycle arrest in G0/G1 (Figure S5 and 5A). However, while PC-1 overexpression results in resistance to apoptosis [21], silencing of NPHP1 resulted in reduced cell viability in response to apoptotic stimuli such as TNFα (Figure 5A, bottom) and increased apoptosis as assayed by TUNEL and cleaved-caspase-3 levels (Figure 5B). Furthermore, silencing NPHP1 *per se* resulted in increased sensitivity to apoptosis in parental MDCK cells (Figure 5C). These data show that PC-1 and NPHP1 are functionally linked to regulate apoptosis, with NPHP1 possibly acting downstream of PC-1. We conclude from these data that PC-1 and NPHP1 cooperate to mediate apoptosis resistance, but not to regulate the cell cycle (Figure 5D).

**Figure 6:**
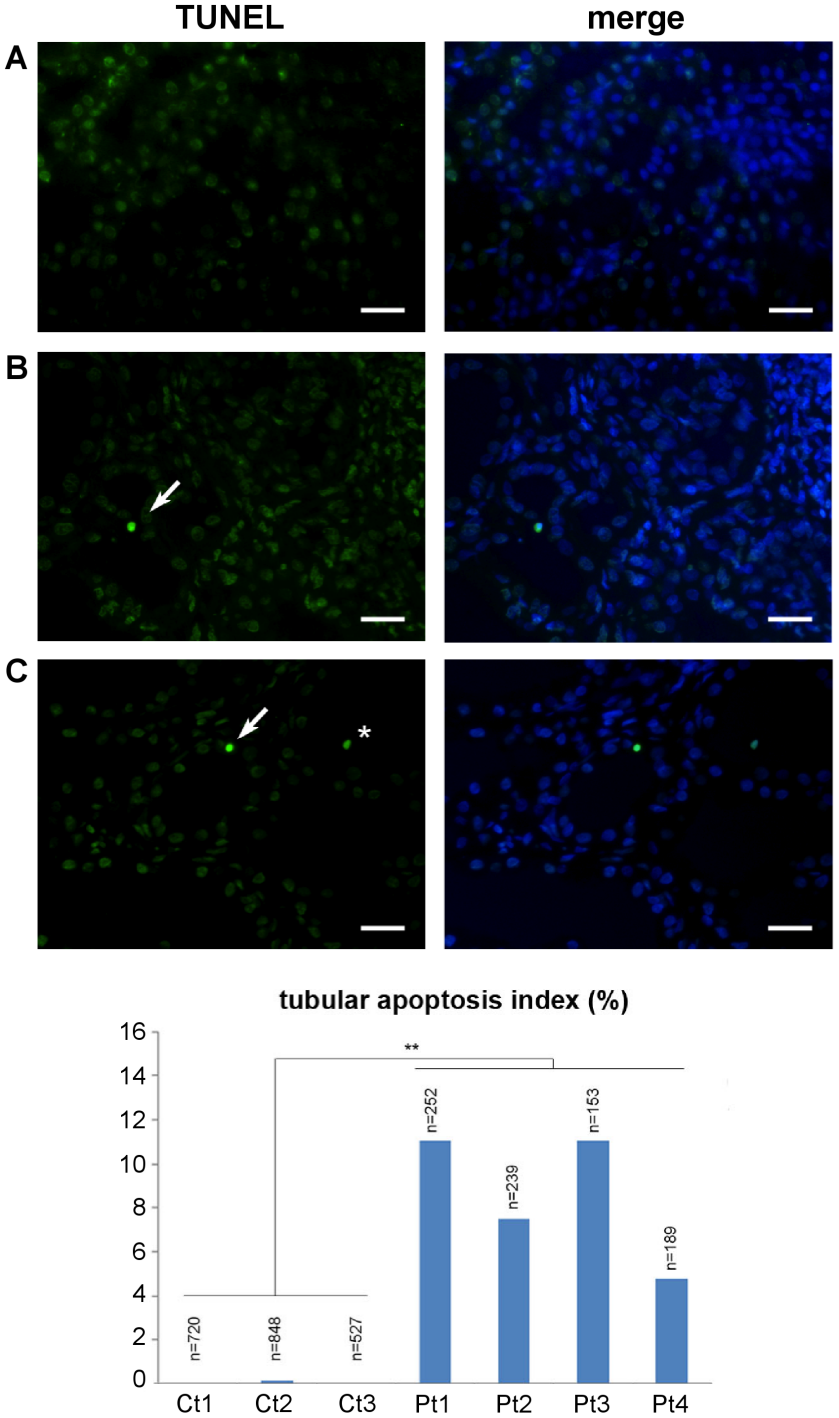
Apoptosis in human kidney tubules of patients mutated for *NPHP1* gene. Apoptosis was determined by the presence of DNA fragmentation, detected by *in situ* TUNEL staining (green) in normal kidneys (**A**; Ct) and in renal specimens from patients carrying homozygous mutations of *NPHP1* (**B–C**; Pt). Co-staining with Hoechst 33342 (blue nuclei) was used to visualize all the cells present in the field (merge). Apoptosis was present in all four patient renal specimens, either in tubular (white arrow) or intra-tubular (asterisk) cells. The total number of tubules evaluated per kidney specimen is indicated on the graph. Controls (Ct) are shown in a linear scale, patients (Pt) in a logaritmic scale. Unpaired t-test with Welch's correction one-tailed was performed for statistical analysis: **p = 0.0057. Scale bar 5 µm.

These results seem to suggest that one important common feature between ADPKD and NPHP might be the susceptibility to apoptosis. Previous studies have indeed described an increased apoptosis in the cystic kidneys of ADPKD patients [23], while the role of apoptosis in NPHP was never investigated before. We therefore performed TUNEL assays on specimens derived from 4 patients affected by NPHP and carrying mutations in the *NPHP1* gene. We found that, indeed, increased apoptosis is a feature of tissues derived from NPHP patients (Figure 6). Collectively, these data suggest that PC-1 and NPHP1 cooperate to regulate the apoptotic response in renal epithelial cells and that this function is likely relevant in the kidney, where loss of either the *PKD1* or *NPHP1* gene function results in increased apoptosis.

## Discussion

In this study we provide evidence for the first time of the existence of a functional polyproline domain within the C-tail of PC-1 able to interact with SH3 domains. In particular, we show that this motif mediates interaction with the SH3 domain of NPHP1 to assemble into a novel, previously unrecognized complex. NMR and ITC experiments supplement this observation, narrowing down the identification of the binding site to the second putative polyproline motif (PP2), which is comprised of residues 4267-4272. The interaction shows a canonical interface between a type II polyproline motif and an SH3 domain, although this relationship is at the low-range strength of interactions.

GST pull-down assays and co-immunoprecipitation studies, however, show that the two proteins assemble specifically into a complex both *in vitro* and *in vivo*. It is possible that the isolated peptides of PC-1 have a lower affinity for the SH3 domain of NPHP1 than they have when embedded in the whole intracellular C-tail. One additional possibility is that other molecules assemble in the complex and help stabilize the interaction. Both PC-1 and NPHP1 exist in multiprotein complexes within cells [3] [8]. Therefore, we cannot exclude the possibility that additional protein-protein interactions (with other partners of the respective complexes) might contribute to stabilizing the complex *in vivo*. One final possibility is that low affinity might be desirable under certain circumstances. Most signaling proteins are expressed in very low amounts in the cell. However, their concentration can be increased when segregated into a local compartment of the cell [24] [25]. In addition, although the interaction is relatively weak, our data clearly demonstrate that abrogation of the polyproline domain/SH3 domain interaction is sufficient to abolish the PC-1/NPHP1 interaction, since mutating the central proline within the polyproline helix (peptPL2) impairs this binding both *in vitro* and *in vivo*. Moreover, the mutation P203L, located in the hydrophobic interaction pocket of NPHP1 SH3 domain, does not affect the domain folding, but totally abrogates the interaction with peptPP2.

Our studies also indicate that a proline to leucine mutation into PP1 (P4172L) decreases the binding of PC-1 C-tail to the SH3 domain of NPHP1, although the isolated peptide containing PP1 (peptPP1) did not cause any peak displacement in 2D ^1^H-^15^N NMR assays. Since the last is a very sensitive and reliable method, these data might suggest that the tertiary structure of the C-tail of PC-1 might be affected by mutations into PP1, which eventually affects the PP2 capability to interact with the SH3 domain of NPHP1. In line with this interpretation, the P4270L mutation is able to completely abrogate the interaction with NPHP1-SH3 both in GST pull-down assays and in the co-immunoprecipitation studies. These results along with the NMR studies with the isolated PP2 and PL2 peptides, strongly suggest that PP2 is indeed the polyproline motif responsible for the interaction. However, we cannot exclude that PP1 can interact with other SH3 domains, even though the low sequence conservation suggests a minor functional role for this motif. Collectively, our results are in perfect agreement with the HUNTER prediction, which showed a very high score for PP2 and a low score for PP1.

Besides the identification of the molecular determinants of this interaction, we believe that our data have important implications for understanding the pathophysiology of renal cystic diseases. In recent years, the ciliary hypothesis of cystogenesis has acquired increasing attention, providing an exciting explanation for why so many different diseases can all result in renal cyst formation [26]. However, it remains to be elucidated whether all of the ciliary proteins, whose gene mutations are known to cause renal cystic diseases, function in a common complex/pathway or whether they independently influence similar cellular functions. A physical and functional interaction was previously reported between genes involved in the two forms of polycystic kidney disease (PKD) (autosomal dominant and autosomal recessive) [27] [28]. Our results show that PC-1, the product of the gene mutated in the most common form of PKD, interacts with NPHP1, the gene product of the *NPHP1* gene, which is mutated in an unrelated cystic kidney disease, nephronophthisis (NPHP). In agreement with our findings, studies performed in the nematode *C. elegans* had suggested that the two proteins might be functionally linked since downregulation of the *PKD1* orthologue *lov-1* or of the *NPHP1* orthologue *nphp1* result in a similar phenotype in this invertebrate model system [29]. However, inactivation of different genes whose products are implicated in ciliary function are indeed expected to generate similar phenotypes, without necessarily implying the association of the proteins involved. Our data uncover the possibility of the existence of a macromolecular complex comprised of several different proteins whose genes are mutated in cystic kidney diseases and warrant future studies on common functions, likely important for preventing renal cystogenesis.

In this study we have shown that one such common function might be the apoptotic response. One intriguing observation that emerges from our data is that PC-1 and NPHP1 cooperate to achieve apoptosis resistance, but do not jointly regulate the cell cycle. PC-1 had been shown to be essential to prevent apoptosis *in vivo* in mice carrying mutations in the *Pkd1* gene [4]. Furthermore, increased apoptosis had also been reported in the cystic kidneys of ADPKD patients [23]. Apoptosis of photoreceptor cells occurs in mice invalidated for *Nphp1*, however no kidney phenotype was associated with the retinal degeneration in those mice [30]. In this study we show that increased apoptosis appears to be a feature of NPHP as well, since analysis of renal specimens from 4 different patients carrying mutations in the *NPHP1* gene revealed the presence of apoptosis in the renal tubules of these tissues. Our data collectively might suggest that the two proteins are functionally linked in renal epithelial cells to protect the renal tubule from apoptosis. How and if this common feature shared by the two diseases might be related to renal cyst formation and/or ciliary function remain to be investigated.

## Materials and Methods

### Antibodies and cell lines employed

Antibodies were obtained as follows: anti-Myc and anti-HA from Roche (1667203 and 1867431 respectively); anti-NPHP1, a kind gift of Dr. G. Walz, University Hospital Freiburg was previously described [12]; anti-phospho and total ERK1/2 antibodies and anti-cleaved caspase-3 (asp 175) from Cell signaling (9102, 9101 and 9661); anti-acetylated tubulin was from Sigma (T6793). horseradish peroxidase-conjugated secondary antibodies from GE Healthcare.

MDCK cells carrying stable transfection of the full-length PKD1 gene were previously described [21]. In brief a vector carrying the zeocine selection cassette was transfected either alone or in combination with the full-length PKD1 gene. Stable transfectants were selected upon exposure to zeocine and individual clones were isolated and characterized for PC-1 expression [21].

### SH3-interaction screen and GST pull-down assays

TranSignal™ SH3 domain arrays by Panomics (Fremont, CA) were screened following the manufacturer's instruction. A HIS-fused construct for expression in bacteria of PC-1 C-tail (aa4132-4303) was generated and expressed in E. coli (BL21 strain). Membranes were incubated with bacterial extract followed by incubation with an anti-HIS antibody. Interactions were visualized by chemiluminescence. Each spot was quantified using the ImageQuant software. The screen was carried out in duplicate using two different preparations of HIS-PC1-CT on two different membrane and generated identical results.

For GST pull-down assays, the indicated GST and HIS-fused proteins were expressed in E.coli strain BL21. Bacteria were lysed and cleaned by centrifugation. Supernatants were mixed and incubated for 2 to 4 h at 4°C, followed by incubation for 2 to 4 h at 4°C with glutathione beads. The beads were centrifuged, washed and proteins were solubilized in Laemmli buffer and analyzed by immunoblotting.

### Sample Preparation for NMR and Binding Assays

The DNA region corresponding to codons 147-212 of the human NPHP1wt and mutant P203L genes was inserted into a GST fusion vector pGEX-2T (Amersham). The constructs were expressed in E.Coli BL21(DE3) and purified as described in [31]. Peptides (peptPP1 = RGSKVSPDVPPPS; peptPL1 = RGSKVSPDVLPPS; peptPP2 = GLRPALPSRLARAS; peptPL2 = GLRPALLSRLARAS; peptPP2short = GLRPALPSRL whose N- and C-termini were acetylated and amidated, were purchased from CASLO (Lyngby, Denmark), their purity was confirmed by HPLC and mass spectrometry. Additional Informations can be found in Materials and Methods S1.

### NMR Measurements and Isothermal Titration Calorimetry (ITC)

Details on NMR titrations, and thermodynamic measurements are described in the supplementary information online. Additional Informations can be found in Materials and Methods S1.

### Molecular docking calculations

Molecular docking calculations of peptPP2 on NPHP1-SH3 (PDB code 1S1N) were performed using the software HADDOCK-2.1, which makes use of biochemical and biophysical information such as chemical-shift perturbation data to drive the docking [18]. Details for docking calculations are described in supplementary informations. Additional Informations can be found in Materials and Methods S1.

### Transient transfections and immunoprecipitation studies

Transient transfections were performed using lipofectamine2000 (Invitrogen). Cells were harvested and lysed for 30 min at 4°C with lysis buffer [250 mM sucrose, 20 mM imidazole, 1 mM EDTA (pH 7.4), and 1% Triton-X100, 0.1% SDS plus Protease Inhibitors Cocktail (Amersham) and phosphatase inhibitors]. Lysates were pre-cleared with the appropriate beads and incubated with high-affinity anti-HA or anti-Myc beads, followed by washing and solubilization in Laemmli buffer for immunoblot analysis.

### Mouse strains and breeding

All the mice used in these experiments were in a C57/Bl6 genetic background; *Pkd1* ^flox/flox^ homozygous mice [16] were crossed with the Ksp Cre mice (kindly provided by Dr. P. Igarashi, Southwestern University, TX-USA) [19] maintained on an heterozygous background as *Pkd1* ^+/−^: Ksp-Cre. After genotyping of the pups, *Pkd1* ^flox/-^: Ksp-Cre mice derived from these crosses were used as experimental animals.

### Immunofluorescence on frozen sections

*Pkd1* ^flox/-^: Ksp-Cre mice were sacrificed at P 4, kidneys were removed and fixed by overnight incubation with 4% paraformaldehyde at 4°C. After washing in PBS, fixed tissues were processed through a graded series of sucrose concentrations from 10–30% in PBS at 4°C, overnight incubation for each step. For better preservation of the cystic structures, kidneys were processed in 30% sucrose- 10% glycerol in PBS solution at 4° for 6 h and then rocked at room temperature for 2 h in a 1∶1 preparation of 30% sucrose in PBS and OCT. Kidneys were then embedded in OCT (Tissue Tek) and frozen in 2- methyl-butane in dry ice.

For immunofluorescence, 10 µm sections were prepared, mounted on slides, air dried for 1 h and washed three times in PBS. Kidney sections were then permeabilized with 0,1% Triton X-100 in PBS for 10 min, blocked with 3% BSA, 5% normal goat serum in PBS for 1 h at room temperature and incubated with primary antibody (NPHP1 1∶100) overnight at 4°C followed by the secondary antibody (Alexa Fluor 488, Molecular Probes, 1∶1000) for 1 h at room temperature; both antibodies were diluted in blocking solution.

Images were obtained with a Perkin Elmer Confocal- UltraVIEW ERS Spinning Disk Confocal.

### Ethical approval of all animal work

All animal care and experimental protocols, including the generation of chimeras, were conducted in accordance with the guidelines provided by the Italian Ministry of Health, upon approval of a specific protocol (IACUC-303) by the institutional care and use ethical committee (I.A.C.U.C.) at the San Raffaele Scientific Institute. Personnel from our own laboratory carried out all aspects of the mouse work under strict guidelines to insure careful, consistent and ethical handling of the mice.

### NPHP1 silencing, cell cycle and apoptosis assays

Silencing of NPHP1 in cells over-expressing PC-1 (employed in Figure 5A, B, Figure S5) were generated by transfection with a MISSION pLKO.1 vector expressing shRNA against a region of the human NPHP1 gene identical to the canine sequence (Sigma-Aldrich, TRCN 0000083822) or the control vector pLKO.1-puro (Sigma-Aldrich, SHC001) and selected with 7.5 µg/ml Puromycin (Sigma-Aldrich, P8833). Several resistant clones were further subcloned and tested for NPHP1 expression levels by western blot. For cell cycle analysis, cells were stained using popidium iodide followed by FACS analysis as previously described [22]. Parental MDCK type II cell lines carrying stable silencing of NPHP1 (Figure 1D and Figure S1B -Ctrl; Figure 5C) were previously described [32]. For apoptosis assays cells were treated with 2 ng/ml recombinant human TNF-α/TNFSF1A (R&D Systems, Minneapolis, MN) and cycloheximide 1 µM. For survival assays, cells were counted using the Countess system (Invitrogen, Carlsbad, CA). For apoptosis assays cells were analyzed by either the DeadEnd Flurometric transferase-mediated dUTP nick-end labeling (TUNEL) system kit (Promega, Madison, WT), or by western blot analysis of cleaved caspase-3. Statistical analysis was performed using the one-way analysis of variance (ANOVA).

### Human renal specimens

Renal specimens from 4 patients with nephronophthisis (1 renal biopsy and 3 kidneys removed at the time of transplantation) fixed in Dubosq-Brazil or formalin had been obtained before DNA screening. Genomic DNA was isolated from peripheral blood by standard methods and analyzed by PCR approach as described previously [33]. All four patients carried homozygous mutations of *NPHP1*. Detection of apoptotic cells was assessed using the *In Situ* cell death detection kit (Roche Applied Science). Three normal renal specimens were used as controls.

### Ethical approval of all work on human specimens

Approval for research on human subjects was obtained from the French ethical committee in accordance with their recommendations (# DGS 950211 obtained in 10/13/94) and after obtaining informed consent from the patients or/and their parents.

## Supporting Information

Figure S1 (A) Hek 293 Cells were co-transfected with full-length Myc-NPHP1 and full-length HA-PC-1. Immunofluorescence analysis was performed using anti-Myc (green) or anti-HA (red) antibodies and counterstained with DAPI (blue) to highlight the nuclei. A partial co-localization in intracellular compartments could be detected as evidenced by the yellow staining in the merge image. Images were captured using a confocal microscope. (B) MEFs derived from a recently described mouse model expressing tagged-endogenous PC-1 (16) were used to immunoprecipitate endogenous Myc-tagged PC-1. Cells expressing the wild-type untagged PC-1 served as a negative control for the immunoprecipitation. Cell lysates from parental MDCK cells (+Ctrl) or NPHP1-silenced cells (-Ctrl) (32) served as a control for NPHP1 western blot. Endogenous NPHP1 and PC-1 co-immunoprecipitated. (C) GST pull-down assays were performed between the SH3 domain of NPHP1 or Abl (negative in the screen in Fig 1B, not shown) fused to GST and the C-terminal tail of PC-1 fused to histidine. NPHP1, but not GST alone or Abl, precipitated the C-terminal tail of PC-1.
(0.42 MB TIF)

Figure S2 (A) 1H-15N HSQC spectra of NPHP1-SH3 with (red) and without peptPP1 (black). The addition of the peptide does not cause any peak displacement indicating that no binding occurs. (B) 1H-15N HSQC spectra of NPHP1-SH3 (black) and NPHP1-SH3-P203L (red). The spectrum of the mutant shows good peak dispersion, indicating that the domain is well folded. (C) 1H-15N HSQC spectra of NPHP1-SH3-P203L with (black) and without (red) peptPP2. The addition of the peptide does not cause any peak displacement, indicating that no binding occurs.
(0.10 MB TIF)

Figure S3 (A) Ribbon representation of a representative structure of the model of NPHP1-SH3 (blue)) in complex with peptPP2 (green sticks). NPHP1-SH3 residues with highest NH chemical shifts are highlighted in red (_Å???>0.1ppm) and pink (0.05<_Å???<0.1). Inter-backbone hydrogen bonds are represented with dotted lines. (B) ITC data for the binding of peptPP2 to NPHP1-SH3. The upper panel shows the sequential heat pulses for peptide-protein binding and the lower panel shows the integrated data, corrected for heat of dilution and fit to a single site binding model using a non-linear least-squares method (line). C. 1H-15N HSQC spectra of NPHP1-SH3 (black) and NPHP1-SH3-P203L (red). The spectrum of the mutant shows good peak dispersion, indicating that the domain is well folded. D. 1H-15N HSQC spectra of NPHP1-SH3-P203L with (black) and without (red) peptPP2. The addition of the peptide does not cause any peak displacement, indicating that no binding occurs.
(0.11 MB TIF)

Figure S4 (A) Immunofluorescence staining of endogenous NPHP1 (green) was performed on previously described *Pkd1^+/+^* (#11) and *Pkd1^−/−^* (#14) Mouse Embryonic Fibroblasts (MEFs) (Distefano et al., 2009). Anti-acetylated tubulin (red) was used as a marker of cilia, whereas DAPI staining (blue) was used to stain the nuclei. NPHP1 can be visualized at the base of cilia both in *Pkd1^+/+^* and *Pkd1^−/−^* (arrowhead). (B) Kidney-specific inactivation of PC-1 using a kidney-specific Cre (Ksp-Cre) system and a floxed Pkd1 mouse model (Wodarczyk et al., 2009) results in polycystic kidney disease. Immunofluorescent analysis of NPHP1 in control (*Pkd1^flox/-^*) and cystic (*Pkd1^flox/-^*:Ksp-Cre) kidneys revealed bright staining in the primary cilium of both normal and cystic tubules. Bar  = 10 µm.
(0.91 MB TIF)

Figure S5 Cell cycle analysis using propidium iodide staining of control (F2) or PC-1 over-expressing (G7/36) MDCK cells showed the typical increase of G0/G1 in cells over-expressing PC-1, not affected by NPHP1 silencing. Statistical analysis was performed using the ANOVA test n.s. not significant.
(0.13 MB TIF)

Table S1 Synthetic peptides used in NMR and ITC titrations.
(0.02 MB DOC)

Materials and Methods S1 Supporting materials and methods.
(0.04 MB DOC)
