# Supplementary figures and images for: Nephrocystin-1 Forms a Complex with Polycystin-1 via a Polyproline Motif/SH3 Domain Interaction and Regulates the Apoptotic Response in Mammals
Source: PLoS One. 2010 Sep 14;5(9):e12719. doi: 10.1371/journal.pone.0012719 (PMC2939065; doi:10.1371/journal.pone.0012719)

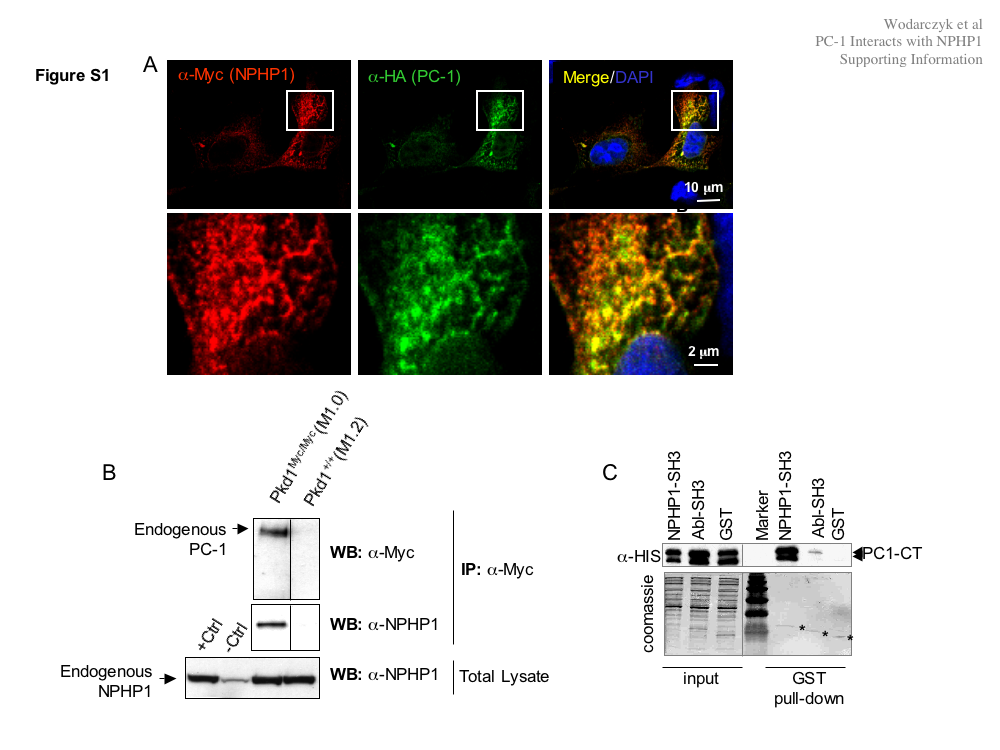

Supplement: Figure S1 — (A) Hek 293 Cells were co-transfected with full-length Myc-NPHP1 and full-length HA-PC-1. Immunofluorescence analysis was performed using anti-Myc (green) or anti-HA (red) antibodies and counterstained with DAPI (blue) to highlight the nuclei. A partial co-localization in intracellular compartments could be detected as evidenced by the yellow staining in the merge image. Images were captured using a confocal microscope. (B) MEFs derived from a recently described mouse model expressing tagged-endogenous PC-1 (16) were used to immunoprecipitate endogenous Myc-tagged PC-1. Cells expressing the wild-type untagged PC-1 served as a negative control for the immunoprecipitation. Cell lysates from parental MDCK cells (+Ctrl) or NPHP1-silenced cells (-Ctrl) (32) served as a control for NPHP1 western blot. Endogenous NPHP1 and PC-1 co-immunoprecipitated. (C) GST pull-down assays were performed between the SH3 domain of NPHP1 or Abl (negative in the screen in Fig 1B, not shown) fused to GST and the C-terminal tail of PC-1 fused to histidine. NPHP1, but not GST alone or Abl, precipitated the C-terminal tail of PC-1. (0.42 MB TIF) [file pone.0012719.s001.tif]

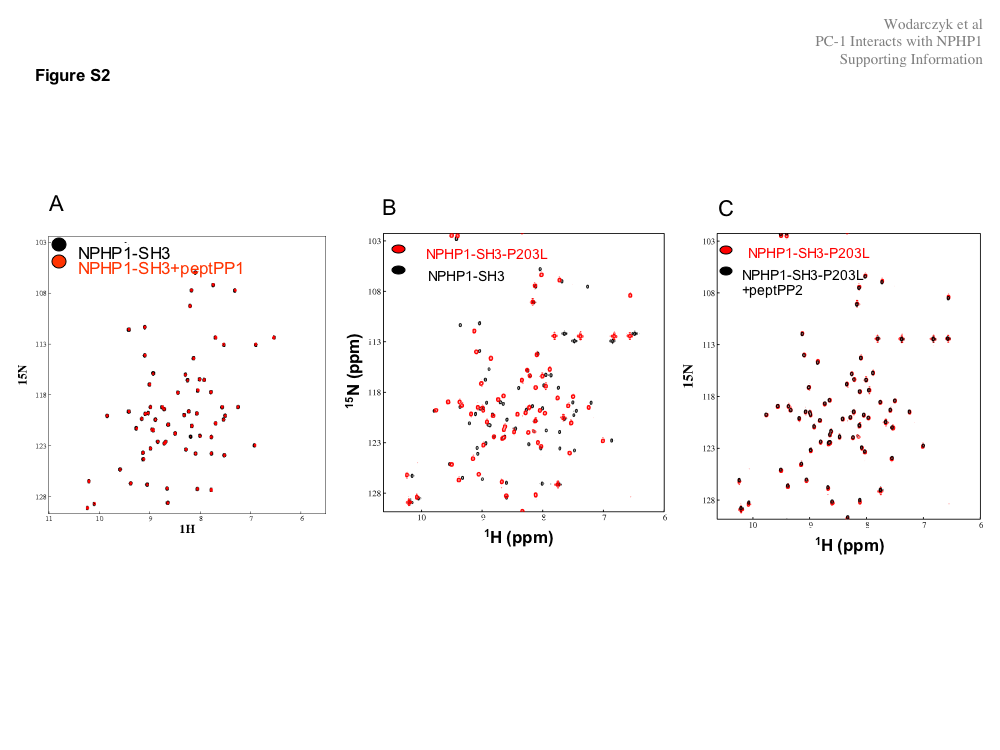

Supplement: Figure S2 — (A) 1H-15N HSQC spectra of NPHP1-SH3 with (red) and without peptPP1 (black). The addition of the peptide does not cause any peak displacement indicating that no binding occurs. (B) 1H-15N HSQC spectra of NPHP1-SH3 (black) and NPHP1-SH3-P203L (red). The spectrum of the mutant shows good peak dispersion, indicating that the domain is well folded. (C) 1H-15N HSQC spectra of NPHP1-SH3-P203L with (black) and without (red) peptPP2. The addition of the peptide does not cause any peak displacement, indicating that no binding occurs. (0.10 MB TIF) [file pone.0012719.s002.tif]

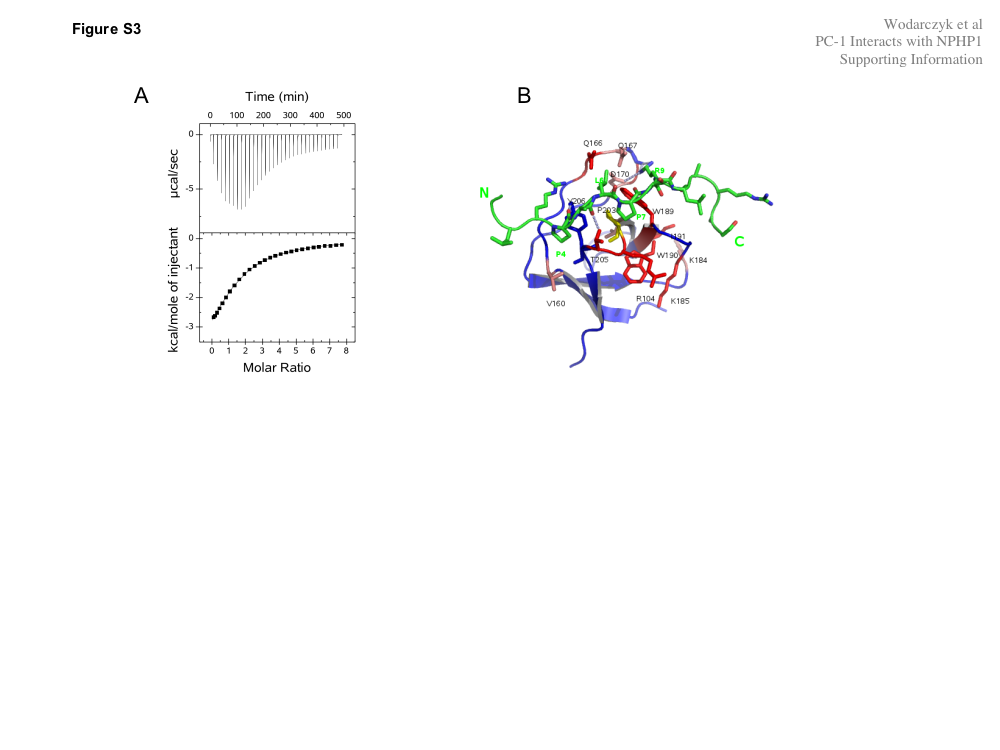

Supplement: Figure S3 — (A) Ribbon representation of a representative structure of the model of NPHP1-SH3 (blue)) in complex with peptPP2 (green sticks). NPHP1-SH3 residues with highest NH chemical shifts are highlighted in red (_Å???>0.1ppm) and pink (0.05<_Å???<0.1). Inter-backbone hydrogen bonds are represented with dotted lines. (B) ITC data for the binding of peptPP2 to NPHP1-SH3. The upper panel shows the sequential heat pulses for peptide-protein binding and the lower panel shows the integrated data, corrected for heat of dilution and fit to a single site binding model using a non-linear least-squares method (line). C. 1H-15N HSQC spectra of NPHP1-SH3 (black) and NPHP1-SH3-P203L (red). The spectrum of the mutant shows good peak dispersion, indicating that the domain is well folded. D. 1H-15N HSQC spectra of NPHP1-SH3-P203L with (black) and without (red) peptPP2. The addition of the peptide does not cause any peak displacement, indicating that no binding occurs. (0.11 MB TIF) [file pone.0012719.s003.tif]

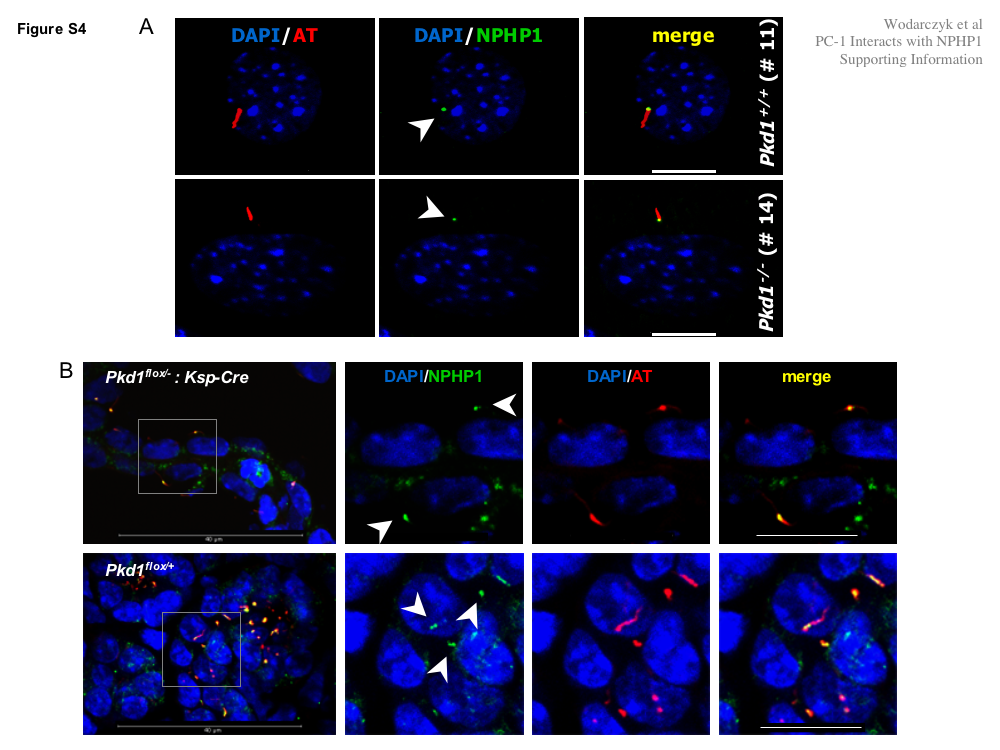

Supplement: Figure S4 — (A) Immunofluorescence staining of endogenous NPHP1 (green) was performed on previously described Pkd1+/+ (#11) and Pkd1−/− (#14) Mouse Embryonic Fibroblasts (MEFs) (Distefano et al., 2009). Anti-acetylated tubulin (red) was used as a marker of cilia, whereas DAPI staining (blue) was used to stain the nuclei. NPHP1 can be visualized at the base of cilia both in Pkd1+/+ and Pkd1−/− (arrowhead). (B) Kidney-specific inactivation of PC-1 using a kidney-specific Cre (Ksp-Cre) system and a floxed Pkd1 mouse model (Wodarczyk et al., 2009) results in polycystic kidney disease. Immunofluorescent analysis of NPHP1 in control (Pkd1flox/-) and cystic (Pkd1flox/-:Ksp-Cre) kidneys revealed bright staining in the primary cilium of both normal and cystic tubules. Bar = 10 µm. (0.91 MB TIF) [file pone.0012719.s004.tif]

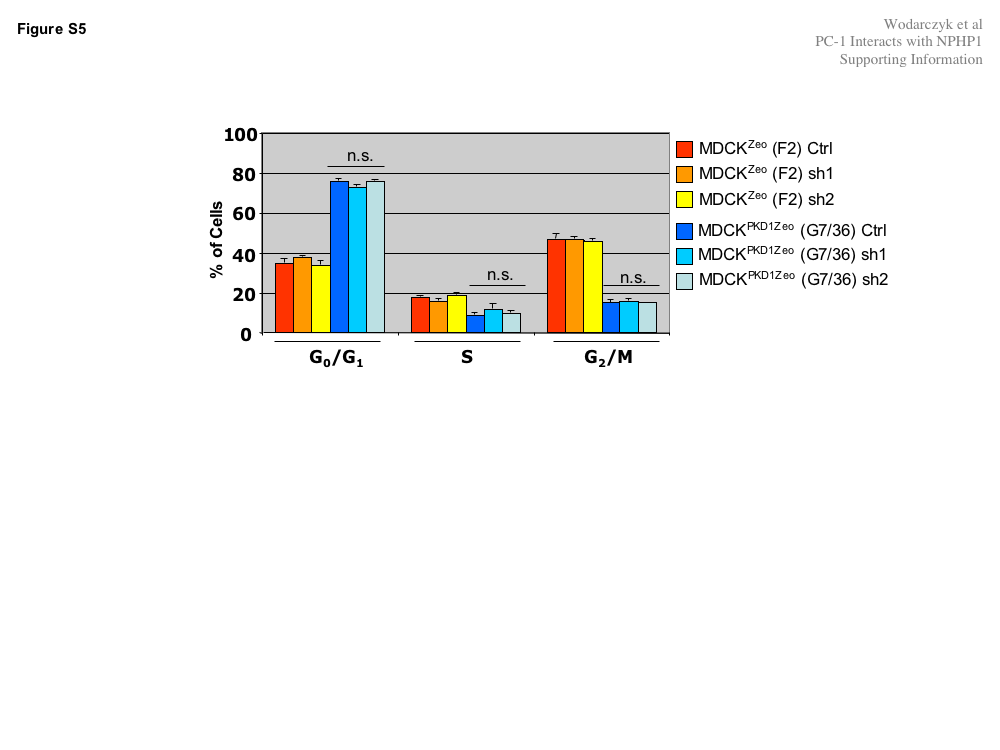

Supplement: Figure S5 — Cell cycle analysis using propidium iodide staining of control (F2) or PC-1 over-expressing (G7/36) MDCK cells showed the typical increase of G0/G1 in cells over-expressing PC-1, not affected by NPHP1 silencing. Statistical analysis was performed using the ANOVA test n.s. not significant. (0.13 MB TIF) [file pone.0012719.s005.tif]
