# Supplementary material for: Nephrocystin-1 Forms a Complex with Polycystin-1 via a Polyproline Motif/SH3 Domain Interaction and Regulates the Apoptotic Response in Mammals
Source: PLoS One. 2010 Sep 14;5(9):e12719. doi: 10.1371/journal.pone.0012719 (PMC2939065; doi:10.1371/journal.pone.0012719)
Supplement: Table S1 — Synthetic peptides used in NMR and ITC titrations. (0.02 MB DOC) [file pone.0012719.s006.doc]

**Table S1.** List of Peptides used in NMR and ITC titrations

| **Peptides** | **Sequence** |
| --- | --- |
| peptPP1 | RGSKVSPDVPPPS |
| peptPP2 | GLRPALPSRLARAS |
| peptPL1(P4172L) | RGSKVSLDVPPPS |
| peptPL2(P4270L) | GLRPALLSRLARAS |
|  |  |
|  |  |
