# Supplementary material for: Nephrocystin-1 Forms a Complex with Polycystin-1 via a Polyproline Motif/SH3 Domain Interaction and Regulates the Apoptotic Response in Mammals
Source: PLoS One. 2010 Sep 14;5(9):e12719. doi: 10.1371/journal.pone.0012719 (PMC2939065; doi:10.1371/journal.pone.0012719)
Supplement: Materials and Methods S1 — Supporting materials and methods. (0.04 MB DOC) [file pone.0012719.s007.doc]

**Supporting Materials and Methods**

**NMR Titrations**

NMR experiments were performed at 25°C on Bruker Avance 600 and 500 MHz spectrometers equipped with triple resonance cryoprobes and pulsed field gradients. Data were processed with NMRPIPE (Delaglio et al, 1995) and analyzed using NMRview (Johnson, 2004). The ^1^H, ^13^C, and ^15^N chemical shifts of the backbone resonances of NPHP1-SH3 were obtained from sensitivity-enhanced three-dimensional HNCA, CBCANH experiments (Sattler et al, 1999). Assignment of the complex NPHP1-SH3/peptPP2 was made by following individual cross-peaks through the titration series.

Typical protein concentration for NMR titration experiments was 0.3 mM in 50 mM phosphate buffer, pH 6.5, 150 mM. In order to minimize dilution and NMR signal loss, titrations were carried out by adding to the protein samples small aliquots of concentrated (15 mM) peptide stock solutions in 50 mM phosphate buffer, 150 mM NaCl, at pH 6.3. For each titration point (typically 0.25, 0.5, 0.75, 1, 1.5, 2, 3, 4, 6, 8, 10 equivalents of ligand) a two-dimensional water-flip-back 15N-edited HSQC spectrum was acquired with 512 (100) complex points, 55 ms (60 ms) acquisition times, apodized by 60 shifted squared (sine) window functions and zero filled to 1024 (512) points for 1H and (15N), respectively. For each residue the weighted average of the ^1^H and ^15^N chemical shifts difference (CSD) was calculated as CSD=[(Δ^2^ HN+Δ^2^N/25)/2]^1/2^ (Grzesiek et al, 1996). Binding constants of peptides to NPHP1-SH3 were estimated monitoring the variation of CSD of individual peaks (8 peaks). Assuming a simple binary reaction between protein and peptide, dissociation constants were obtained from least squares fitting of CSD as a function of total ligand concentration according to the relation (eq. 1):

*
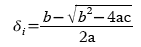
* (1)

with a=(K_a_/δ_b_) [P_t_], b=1+K_a_([L_ti_]+[P_t_]), and c=δ_b_K_a_[L_ti_], where δ*_i_* is the absolute change in chemical shift for each titration point, [L_ti_] is the total ligand concentration at each titration point, [P_t_] is the total protein concentration, K_a_=1/K_d_ is the binding constant, and δ_b_ is the chemical shift of the resonance in the complex. K_d_ and δ_b_ were used as fitting parameters using the Xmgrace program.

**Isothermal Titration Calorimetry (ITC) thermodynamic analysis**

Thermodynamic analyses were performed at 21 °C using a VP-ITC isothermal titration calorimeter (MicroCal LLC, Northampton, MA, USA). Peptides (15 mM) were injected in NPHP1-SH3 solutions (0.38 mM). The reaction buffer contained 50 mM phosphate buffer, pH6.5. Titrations were performed using stirring of 307 rpm, with 33 injections, up to 10 μL for each injection and 15 minutes of spacing between injections into the ITC sample cell of 1.41 ml. Control experiments were performed under identical conditions to determine the dilution heat of the titrant peptide into buffer and of the buffer into protein samples. The final data were analyzed using the software ORIGIN 7.0 (OriginLab Corp., Northampton, MA, USA) integrals of differential heats of injection were corrected by dilution heats and finally fitted by one-site binding model and nonlinear least square regression. Measurements were performed in duplicate. The list of peptides used in the NMR and ITC titrations is summarized in Table S1.

**Molecular docking calculations**

Molecular docking calculations of peptPP2 on NPHP1-SH3 (PDB code 1S1N) were performed using the software HADDOCK-2.1(de Vries et al, 2007). PeptPP2 was build using Pymol software (DeLano Scientific LLC) and using as template the peptide from 1CKA SH3-complex crystal structure, so that residues comprising the sequence PALP 5-8 adopted a type II polyproline helix conformation. Amino and amidic groups were used to block the N- and C-terminus of peptPP2. An ensemble of 10 structures for both SH3 protein and peptPP2 extracted from a short molecular dynamics simulations performed with GROMACS4-05 (1ns) was used for docking calculations. The HADDOCK protocol follows a three-stage docking procedure which includes (1) randomization of orientations and rigid body minimization, (2) simulated annealing in torsion angle space, and (3) refinement in Cartesian space with explicit water. Residues showing chemical shifts larger than the average chemical-shift difference plus one standard deviation (Q166, Q167, K185, W189, L201, P203, T205, and for peptPP2: P5, A6, L7, P8, R10) were used as ambiguous interaction restrains (AIRs). Residues that were close to the threshold, or with small (<50%) solvent-accessible surface areas, were not included in AIRs. The P203 was also included in the AIRs, as mutation of this residue impairs binding. Optimized parameters for liquid simulation (OPLS) were used for the protein (parallhdg5.3.pro and topallhdg5.3.pro). During the rigid body docking, 1000 structures were calculated, allowing the ligand to explore solutions rotated by 180 degrees, thus increasing the sampling of the solutions. The best 200 solutions in terms of HADDOCK score were selected for a semiflexible simulated annealing, followed by water refinement, in which the side-chains of SH3 and of peptPP2 located at the binding interface (SH3: residues 165-170, 184-190, 200-206; peptPP2 residues 2-4, 9-15) were allowed to move in a semi-rigid-body docking protocol to search for conformational rearrangements. The analysis of the simulations was performed applying in-house python and tcl scripts. Root-mean-square deviations (rmsd) were calculated using the VMD program (http://www.ks.uiuc.edu/Research/vmd/). The fitting of the protein was performed on the flexible residues (165-170, 184-190, 200-206) using the Kabsch algorithm (Kabsch, 1978); the rmsd of peptPP2 was calculated on the backbone of residues 5 to 8. The final rmsd matrix was then clustered using the algorithm described in Daura et al (Daura et al, 1999), where a cluster is defined as an ensemble of at least two conformations displaying an rmsd smaller than 2.0 Å. The final structures were clustered and scored using a combination of energy terms defined as: 1.0*EvdW + 1.0*Eelec + 0.1*EAIR +1.0*Edesolv, the subscripts denoting: vdW, the van der Waals energy; elec, the electrostatic energy; AIR, the ambiguous interaction restraint energy; and desolv, the desolvation energy calculated using the atomic desolvation parameters as described in (Fernandez-Recio et al, 2004).

**Supporting References**

Daura X, Antes I, van Gunsteren WF, Thiel W, & Mark AE (1999) The effect of motional averaging on the calculation of NMR-derived structural properties. *Proteins* **36**: 542-555

Delaglio F, et al. (1995) NMRPipe: a multidimensional spectral processing system based on UNIX pipes *J Biomol NMR* 6**:** 277-93.

Distefano G, et al. (2009) Polycystin-1 regulates extracellular signal-regulated kinase-dependent phosphorylation of tuberin to control cell size through mTOR and its downstream effectors S6K and 4EBP1 *Mol Cell Biol* 29**:** 2359-71.

Fernandez-Recio J, Totrov M, & Abagyan R (2004) Identification of protein-protein interaction sites from docking energy landscapes. *J Mol Biol* **335**: 843-865

Grzesiek S, Stahl SJ, Wingfield PT, Bax A (1996). The CD4 determinant for downregulation by HIV-1 Nef directly binds to Nef. Mapping of the Nef binding surface by NMR. Biochemistry. 35:10256-61.

Johnson BA (2004) Using NMRView to visualize and analyze the NMR spectra of macromolecules *Methods Mol Biol* 278**:** 313-52.

Kabsch W (1978) A discussion of the solution for the best rotation to relate two sets of vectors. *Acta Crystallographica Section A* 34: 827-882.

Sattler M, Schleucher, J, Griesinger, C (1999) Heteronuclear multidimensional NMR experiments for the structure determination of proteins in solution employing pulsed field gradients *Prog NMR Spectroscopy* 34**:** 93-158.
